# XLPE and Beyond: A Review of Recent Progress in Polymer Nanocomposites for Dielectric Insulation in High-Voltage Cables

**DOI:** 10.3390/ma18245553

**Published:** 2025-12-10

**Authors:** Alexander A. Yurov, Ivan N. Zubkov, Alexey V. Lukonin, Oleg Y. Kaun, Alexander E. Bogachev, Victor A. Klushin

**Affiliations:** 1 Department of Digital Technologies and Platforms in Electric Power Industry, Don State Technical University, Gagarin Square 1, 344003 Rostov-on-Don, Russia; ayurov@donstu.ru (A.A.Y.); alukonin@donstu.ru (A.V.L.); okaun@donstu.ru (O.Y.K.); abogachev@donstu.ru (A.E.B.); 2 Resource Center for Collective Use of the Scientific and Educational Center “Materials”, Don State Technical University, Gagarin Square 1, 344003 Rostov-on-Don, Russia; ivan.n.zubkov@yandex.ru

**Keywords:** crosslinked polyethylene, nanofillers, dielectric insulation, space charge accumulation, breakdown voltage strength

## Abstract

Crosslinked polyethylene (XLPE) has been the cornerstone material in the power industry for insulating high-voltage cables due to its exceptional properties, including reduced dielectric loss, high dielectric constant and thermal conductivity, and excellent resistance to electrical stress. In the current study, in order to further enhance the electrical and mechanical properties of XLPE’s various types of nanofillers such as metal oxides, boron nitride nanosheets of nanosilica and graphene oxide are incorporated into the XLPE matrix. These nanoparticles promote the occurrence of numerous trap sites, even at modest concentrations, due to their extensive interfacial regions, which affect crucial characteristics including breakdown voltage strength, electrical tree growth, structural defects, space charge accumulation, and thermal aging. The present review summarizes the effects of nanoparticles on the dielectric performance of XLPE. At the same time, the current advancements in the development of a new generation of recyclable insulation materials are briefly discussed.

## 1. Introduction

**Figure 1:**
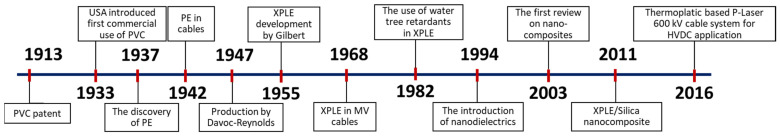
Progress in the development of polymer insulation for cables throughout history.

With the ever-increasing shift towards renewable energy, and large-scale urbanization taking place across the developing world, the power industry is witnessing significant development and modernization, with the aim of making grid systems cost-effective, while at the same time reducing the power loss that takes place during transmission over long distances [1,2]. Polymer insulators are indispensable to power grid systems, and as the aging infrastructure struggles under rising voltage stresses, polymer composites, beyond just insulation, facilitate smarter, tougher, and more thermally resilient power cables [3,4]. One of the earliest known uses of insulated cables can be traced back to 1881, when Thomas A. Edison developed a power distribution system by deploying copper rods insulated in jute and placing them inside iron pipes filled with wax. However, it was only 9 years after Thomas A. Edison’s work, in 1890, when Dr Ferranti implemented a high-voltage insulated conductor in the world’s first modern power station for the London Electric Supply Corporation, supplying high-voltage alternating current (HVAC) of ~10 kV [5]. While there are records of fluid/oil-impregnated paper cables going back as far as 1872, it was only at the beginning of the 20th century when fully established designs emerged. However, they had a number of drawbacks, including large dielectric loss and expensive operation, resulting in poor insulation materials. At the same time, insulation materials based on natural rubber and resins have also been deployed as insulators, but their limitations in terms of performance led to the development of synthetic materials [6]. Polymer insulators were first introduced in the early 1930s as a viable replacement for ceramic and glass insulators, and have since gained popularity due to their technical advantages, including excellent electrical properties, high strength-to-weight ratio, and manufacturing ease [7,8]. Figure 1 highlights the progress in polymer insulation since the beginning of the 20th century [9].

At present, various synthetic polymers, such as epoxy resin, polyethylene (PE), polypropylene, crosslinked polyethylene (XLPE), polyvinyl chloride (PVC), polymethyl methacrylate, polycarbonate, polyamide, polyimide, and silicon rubber, have found large-scale application in the electrical power industry [10,11]. Among them, XLPE is by far the most favored material and has been the mainstay of HVAC cable manufacturing industries for the last 4 to 5 decades. In recent times, high-voltage direct current (HVDC) cable technology has shown increasing use of XLPE-based insulation, as demonstrated by NKT’s launch of a 640 kV XLPE-based HVDC cable capable of transmitting at least 3 GW [12]. Cross-linking enhances PE’s thermal stability under load, which in turn ensures the preservation of functional properties at high temperatures, as well as reducing the shrinkage of insulating material. As a result, XLPE’s superior performance positions it ahead of other polymer materials as an insulating material for conductors, as it does not contract when subjected to heat generated by conductors [13,14]. While the capabilities of conventional XLPE have already reached a threshold in terms of purity and thermal stability, for manufacturing insulation with higher operating temperatures, two different approaches have been adopted: (i) using nanocomposites based on XLPE, PE, and organic/inorganic fillers [15,16,17,18], and (ii) replacing XLPE with copolymers of PP, ethylene, and propylene or terpolymers of ethylene and propylene coupled with a diene component [19,20,21]. The technology of XLPE-based power cable insulations has been thoroughly studied and implemented successfully by manufacturers around the world and the introduction of nanofillers such as SiO_2_, Al_2_O_3_, TiO_2_, carbon black, graphene, graphene oxide, etc., for the manufacture of power cable insulation has driven the interest of scientific as well as industrial societies with aim of creating an XLPE-based polymer composite with significantly lower electrical conductivity and loss factor, higher dielectric breakdown strength, an absence of electric treeing formation, and higher operating temperature stability.

At the same time, the for HVDC cables, XLPE-based insulation has several limitations including a relatively low working temperature which restricts the power capacity of HVDC cables; on the other hand, the thermosetting nature of XLPE also presents a serious problem in terms of material recycling and environmental pollution reduction [22,23,24,25]. Currently, research on HVDC cables is undergoing a transition to develop advanced cables that will perform well beyond the limits of XLPE. Thermoplastic polymers are the most extensively studied materials in terms of the insulation of HVDC cables. In contrast to traditional XLPE insulators, thermoplastic polymer-based insulators are free of crosslinking byproducts and exhibit superior electrical and mechanical qualities, attributable to their high breaking strength, flexibility, and thermal resistance [26,27,28,29,30]. HVDC cable transmission is considered an efficient and environmentally viable way for accessing remote large-scale renewable energy sources (such as offshore wind or desert photovoltaics), the integration of bulk renewable power into the grid, and providing a power backbone for interconnected AC systems, making HVDC cable transmission a key enabler of decarbonization [31,32]. HVDC cable technologies also contribute to the global United Nations Sustainable Development Goals (SDGs), namely SDG 7 “Affordable and clean energy” and SDG 13 “Climate action”.

The present review is focused on highlighting the recent progress made in the materials used for designing of polymer composite-based insulation for high-voltage cables with the primary focus on XLPE-based composite materials with critical factors, which could influence electrical performance under stress, while also giving insights into advancements in the development of a new generation of recyclable insulation materials for HVDC cables.

## 2. Crosslinked Polyethylene (XLPE)

XLPE is produced by crosslinking low-density PE (LDPE) to improve its thermo-chemical resistance and mechanical qualities. As a result of the crosslinking of PE chains, the operational temperature of XLPE increases to 90 °C, in contrast to LDPE which has an operational temperature of about 65–70 °C. There are reports of XLPE working stably at a temperature of 130 °C for about 36 h; however, if the temperature increases to 250 °C (for example, during a short circuit), the XLPE destructs within seconds [33].

**Figure 2:**
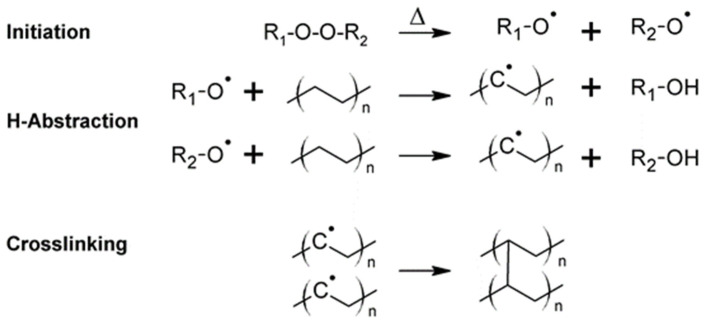
Thermal curing of LDPE with dicumyl peroxide. Redrawn and adapted from reference [41].

The crosslinking of LDPE can be carried out using physical (electron irradiation) and chemical methods, with the latter being preferred in the cable industry due to its simplicity and there being no requirement of high-cost equipment. The chemical crosslinking is carried out by the addition of radical initiators such as dicumyl peroxide or 2,5-bis-(tert-butylperoxy)-2,5-dimethylhexane, which undergo homolytic bond cleavage during the extrusion process to initiate the crosslinking [34] (see Figure 2).

As a result of the bond cleavage of dicumyl peroxide various by-products are formed including CH_4_, acetophenone, and cumyl alcohol. In order to avoid the formation of voids from gaseous by-products, the curing of the extruded material is carried out at elevated pressures after which the extruded XLPE is transferred to an oven at a temperature of 70 °C to eliminate by-products [34].

**Figure 3:**
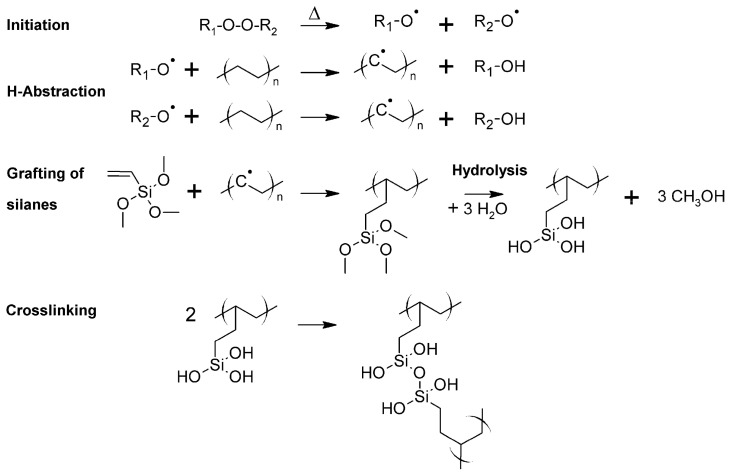
Radical grafting of alkoxysilane crosslinkers onto polyethylene using dicumyl peroxide followed by catalyzed hydrolysis of alkoxysilane to obtain crosslinked PE. Redrawn and adapted from reference [41].

Another commonly employed method is the crosslinking of a chemically modified PE in the presence of silane coupling agents (the silane method). The grafting of silane onto the main PE chain is ensued by the vinyl group. The hydrolysable alkoxy groups present in the PE react with water or moisture, creating a three-dimensional network of siloxane linkages. The grafting of alkoxysilane onto the PE chain occurs through radical formation (Figure 3).

Further, the grafted alkoxy groups interact with water/moisture and a catalyst to form silanol groups [35,36,37], which are further subject to a condensation reaction resulting in a crosslinked polymer network (Figure 3).

The crosslinking of LDPE is also carried out using ionizing methods such as an electron beam or gamma radiation generated from a Co^60^ source [38,39,40]. The crosslinking takes place through a free radical mechanism, which involves the extraction of a hydrogen radical from the polymer chain with accelerated electrons or with an electromagnetic wave, leading to the formation of radicals in the PE chains, which in turn recombine and result in crosslink sites [41].

The degree of crosslinking can be controlled by varying the concentration of peroxide, and the intensity of the ionizing energy. In general, the degree of crosslinking is increased by the addition of sensitizers such as acrylates during the extrusion process. After the insulating layer is extruded under ambient conditions, the crosslinking process is completed. In order to deter an uncontrolled increase in temperature during crosslinking, the extruded cables are exposed to radiation several times until the targeted crosslinking is attained [42].

## 3. Nanocomposite-Based XLPE Insulation

The insulating material in high-voltage transmission cables is continuously subjected to electrical and thermal stresses, which leads to the deterioration of its performance [43]. Under a full operational load, high-energy electrons tend to accumulate within the insulator, forming space charges, which results in PD, a phenomenon characterized by localized electrical discharges within the insulation system. These discharges eventually lead to the breakdown of insulation materials [44].

Additives play a crucial role in countering breakdown and failures in the insulation system. The introduction of nanoparticles, including metal and non-metal oxides/carbides and different types of graphite, into insulator materials is a widely implemented method for improving the properties of insulation materials. There are four types of nanofillers based on their dimensional geometries [45]:

- Zero-dimensional—nanoparticles of SiO_2_, TiO_2_, nanoclusters, and fullerenes;
- One-dimensional—plates, laminas, and shells;
- Two-dimensional—nanotubes and nanofibers;
- Three-dimensional—spherical nanoparticles.

The choice of nanoparticles depend on the desired mechanical, thermal, and, more importantly, electrical properties of the insulation material. For example, it has been reported in [46,47] that carbon nanotubes could be used to increase the electrical and thermal resistivity of an insulation material, and that Al_2_O_3_ can be used to obtain enhanced thermal conductivity, while TiO_2_ nanoparticles are used to induce photocatalytic properties. BN is added to the polymer matrix to improve the material’s mechanical strength, hardness, corrosion resistance, and non-linear electrical behavior.

### 3.1. Metal Oxide-Based XLPE Insulation Materials

Metal oxide-based nanofillers are often incorporated in the polymer matrix to enhance its electrical, thermal and mechanical properties. Al_2_O_3_ is commonly deployed thanks to its low cost and high electrical resistivity and thermal conductivity. It has been reported by the authors of the work [48] that mixing Al_2_O_3_ with XLPE could reduce the ignition and heat release rates. T J Mohamed et al. [49] studied the influence of Al_2_O_3_ loading on electrical tree propagation in XLPE. It was reported that the value of the electrical tree inception voltage increased from 11.2 kV for pure XLPE to 14.8 kV for XLPE modified with 1 wt.% Al_2_O_3_, while the time required for treeing to reach 2 mm, increased from 21 h (pure) to 23 h (modified with 1 wt.% Al_2_O_3_), and the insulation breakdown time was improved by 13%.

Nurul Razak et al. [50], in their investigations, studied the influence of incorporating hybridized layered double hydroxide (LDH) with an Al_2_O_3_ nanofiller into the XLPE matrix. The XLPE/LDH-Al_2_O_3_ nanocomposites showed enhanced mechanical, thermal, and dielectric properties, including PD resistance, AC breakdown strength, and tensile properties. At the same time, the value of the PD was reduced by 52.2% and the AC breakdown strength increased by 15.6% compared to pure XLPE; meanwhile, the tensile strength, Young’s modulus, and elongation at break of the XLPE/LDH-Al_2_O_3_ nanocomposites were enhanced by 14.4%, 31.7%, and 23%, respectively. At the same time, the effectiveness of the insulation material was analyzed in terms of its PD magnitude parameter, with pure XLPE showing the highest PD magnitude. Increasing the loading of LDH-Al_2_O_3_ from 0.2 to 0.8 wt.% resulted in a steady reduction in PD magnitude. However, when the nanofiller loading was increased to 1.0 wt.%, a sharp increment in the PD magnitude was recorded. The authors associated this increment in the PD magnitude to the poor distribution of nanoparticles in the polymer matrix creating voids. Introducing LDH-Al_2_O_3_ nanoparticles into the XLPE matrix also led to an increase in the AC breakdown strength of the insulating materials. The breakdown strength increased from 169.38 kV/mm for pure XLPE to 194.78 kV/mm for XLPE modified with 0.8 wt.% LDH-Al_2_O_3_.

Chen Daoyuan et al. [51] in their work reported the influence of thermo-oxidative aging on the space charge characteristics and physicochemical properties of pure XLPE and XLPE/Al_2_O_3_ nanocomposites for HVDC cables. The XLPE/Al_2_O_3_-A showed an excellent space charge distribution and physicochemical properties when aged for 672 h in comparison with pure XLPE. While in the case of XLPE/Al_2_O_3_-B, hetero charge accumulation near the electrode was registered after aging for 336 h. The accumulation of hetero charge increased with the aging time leading to an increase in the field strength distortion. However, the authors did not reveal the content of the Al_2_O_3_ nanofiller for commercial reasons.

In the work [52], the effect of KH550-functionalized α-Al_2_O_3_ nanosheets on the DC breakdown strength and space charge accumulation in XLPE/α-Al_2_O_3_ nanocomposites was studied. It was reported that the addition of KH550-functionalized α-Al_2_O_3_ significantly improved the DC breakdown strength of the nanocomposites. Upon increasing the content of functionalized α-Al_2_O_3_ from 0.2 to 1.0 wt.%, a sharp increase in the breakdown strength was observed. The maximum DC breakdown strength (320 kV/mm) was shown by the nanocomposite containing 1.0 wt.% of KH550-functionalized α-Al_2_O_3_; in comparison, the pure XLPE had a breakdown strength of only about 220 kV/mm.

Another commonly used nanofiller for XLPE-based insulation cables is ZnO, which due to its high thermal conductivity and non-linear current–voltage behavior is considered an important electric stress control material. The non-linear conductivity of ZnO is a result of various phenomena, including grain boundary effects, voids in the lattice, and polarization under the external electric field, which in turn could induce improved dielectric performance in the nanocomposites [53].

Eldesoky et al. [54] prepared XLPE-based nanocomposites modified with functionalized/non-functionalized ZnO (loading varied from 0.5 to 5 wt.%) using the melt blending method. The incorporation of ZnO nanoparticles into the polymer matrix resulted in improved electrical properties (see Table 1) in comparison with pure XLPE.

As can be seen from Table 1, the AC breakdown strength of XLPE/functionalized ZnO nanocomposites is higher than that of the non-functionalized one. In addition, upon increasing the concentration of functionalized ZnO up to 3.5 wt.%, the AC breakdown strength increases, while at higher concentrations the agglomeration of nanoparticles occurs causing a reduction in the breakdown strength.

At the same time, the XLPE–non-functionalized ZnO sample showed the highest value of relative permittivity, alongside a high dielectric loss. The authors explained this by focusing on the reduced interface interaction area between the polymer chains and ZnO which caused nanoparticle agglomeration. The agglomerated nanoparticles in turn acted as voids between the XLPE chains and led to their mobility, and resulted in more molecular polarization. From Table 1, it can be seen that XLPE–functionalized ZnO with 2 wt.% has the lowest relative permittivity value, which is about 4.1% less compared to pure XLPE at a power frequency of 50 Hz. The obtained results indicate that the optimal loading of functionalized ZnO in the polymer matrix was 2 wt.%.

In the study carried out by Mansor et al. [55], the electrical treeing characteristics of XLPE–functionalized ZnO nanocomposites were compared with pure XLPE. It was found that the addition of (3-aminopropyl)triethoxysilane functionalized ZnO nanofillers to XLPE did not improve the tree inception voltage; in fact, upon increasing the loading of functionalized ZnO from 0.5 to 1.5 wt.%, the tree inception voltage decreased to 9 kV in contrast to 15.7 kV for pure XLPE. Conversly, the nanocomposites with 1.0 wt.% functionalized ZnO led to a ~14% increase in the tree growth time compared with pure XLPE.

In another study carried out by the same group, the effect of the addition of non-functionalized ZnO nanofiller to XLPE on the electrical tree formation behavior was studied [56]. Upon the addition of 0.5 wt.% nanofiller to XLPE, the tree inception voltage improved by almost 7.7%; meanwhile, increasing the loading of nanofiller to 1.0 wt.%, a 14.7% improvement in comparison with pure XLPE was observed. However, increasing the nanofiller loading to 1.5 wt% exhibited only a slight increase in the electrical tree growth propagation trend compared with the other nanofiller concentrations, possibly due to the ZnO agglomeration and large void formation at the interface or trapped regions.

TiO_2_ is a widely used nanofiller material in the power industry that improves the electrical and thermal insulation properties of polymers due to its high relative permittivity. Essawi et al. [57] studied the influence of various loadings of TiO_2_ nanoparticles (1.0–7.0 wt.%) on the dielectric and mechanical properties of XLPE/TiO_2_ nanocomposites. The effect of temperature on the AC dielectric strength of pure XLPE and XLPE/TiO_2_ nanocomposites was studied by connecting the test sample to a transformer (50 Hz, 220 V/100 kV) under various temperatures. It was shown that the AC breakdown voltage increased significantly under all of the temperature regimes, when the loading of the TiO_2_ in the nanocomposite was 5 wt.%. The XLPE nanocomposite with 5 wt.% TiO_2_ showed the maximum tensile strength value (10.89 MPa) and elongation at break (310.91%) compared to pure XLPE.

Abd Rahman et al. [58] also studied the effect of the addition of TiO_2_ on the space charge accumulation and dielectric breakdown strength of XLPE and made a comparison between TiO_2_- and BaTiO_3_-based XLPE nanocomposites. However, it was reported that the AC breakdown strength of XLPE increases only upon the addition of BaTiO_3_ while the TiO_2_ caused a reduction. This can be due to the poor dispersion of TiO_2_ causing agglomeration or due to the use of a noncompatible form of TiO_2_ by the authors. The work does not satisfactorily describe the materials used to prepare the test samples.

The authors of the work [59] showed that XLPE nanocomposites based on amino silane-functionalized TiO_2_ nanoparticles had an improved relative permittivity, AC breakdown strength, and dielectric loss value, with the optimal results shown by the nanocomposite with a functionalized TiO_2_ loading of 2.0 wt.%. While the group of Wang et al. [60] reported a suppressed homocharge injection, enhanced DC dielectric strength, increased crystallinity, and changed morphology when TiO_2_ was functionalized with the silane coupling agent KH560. The XLPE nanocomposites based on KH560-functionalized TiO_2_ showed a 13.5% increase in the DC breakdown strength compared to pure XLPE. The improved space charge characteristics and the increased dielectric strength were a result of the incorporation of nano-TiO_2_ which created a potential barrier and hindered the transport of charge carriers during the charge movement. In the case of the proper dispersion of nanoparticles, the interaction zone plays an important role in suppressing charge transport. When the concentration of nanoparticles increases, the space between neighboring particles reduces, which in turn leads to overlapped interphase regions, which could then lead to relatively shallower traps and form paths with relatively higher charge carrier mobility among potential barriers.

### 3.2. Boron Nitride-Based XLPE Insulation Materials

In recent years, boron nitride (BN) has been gaining popularity among researchers as a potential additive for insulation in the power industry given its ability to enhance both thermal conductivity and electrical insulation properties [61]. Adding BN to insulation materials can improve the materials’ ability to dissipate heat and resist electrical breakdown [62]. Wang et al. [63] studied the influence of doping nanohexagonal boron nitride (*h*-BN) on the DC breakdown strength and space charge properties of pure XLPE. The XLPE/*h*-BN nanocomposites doped with 0.1 wt.% nano-*h*-BN showed an increase in the breakdown strength by 39% compared to pure XLPE. The thermally stimulated current results confirmed the formation of deep charge traps (above 1 eV) in the XLPE matrix as a result of *h*-BN doping, which could in turn be the primary reason for the improved DC breakdown strength. At the same time, the XRD curves of the XLPE and XLPE/*h*-BN samples showed no differences, indicating that *h*-BN does not introduce impurities or a change in the crystallinity of XLPE.

**Figure 4:**
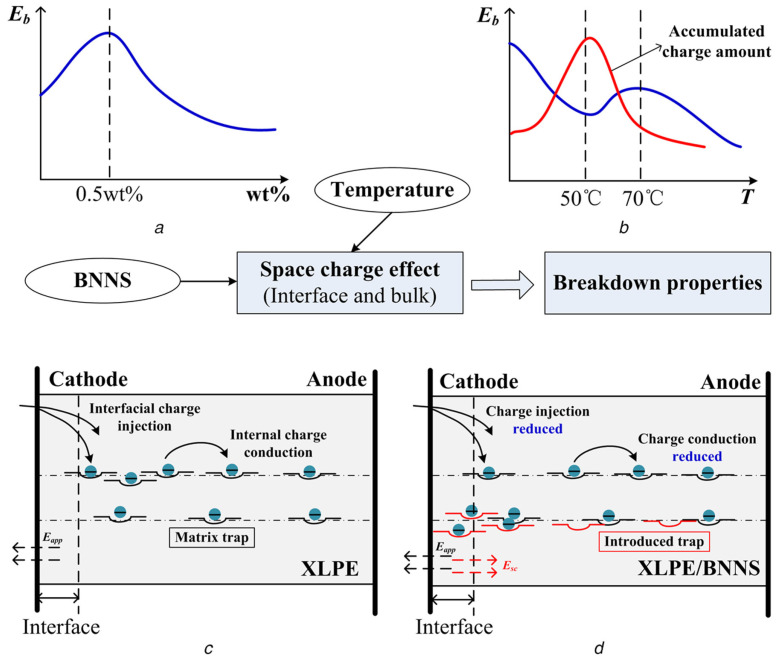
Logical diagram of functionalized BNNS concentration, temperature, and space charge versus breakdown strength. (**a**) DC breakdown strength versus BNNS concentration, (**b**) breakdown strength and accumulated charge amount versus temperature, (**c**) charge transport in pure XLPE, and (**d**) charge transport in XLPE–functionalized BNNSs [65].

In another study conducted by Zhou et al. [64], BN nanoparticles (BNNPs) and BN nanosheets (BNNSs) were used as nanofillers to investigate the effect of nanofiller concentration and morphology on the electrical properties of pure XLPE. It was reported that the relative permittivity of the XLPE/BNNP and XLPE/BNNS nanocomposites was about 11% and 9% higher compared to pure XLPE when the nanofiller content was 1.0 wt.%. At the same time, the DC breakdown strength of the composite reached a maximum when the concentration of nanofiller was 0.5 wt.%. Nanocomposites based on BNNSs showed a 33% increase in the DC breakdown strength compared to pure XLPE; however, in the case of the BNNP nanofiller, the DC breakdown strength was only 13% higher compared to pure XLPE. Based on the results it was concluded by the authors that BNNSs could significantly improve the dielectric and DC breakdown properties of pure XLPE. Li et al. [65] investigated BNNSs functionalized with KH550 as a nanofiller for pure XLPE. In their work the primary focus was centered around studying the influence of functionalized BNNSs on space charge and DC breakdown strength at various temperatures. The DC breakdown strength of the composite reached its maximum when the concentration of nanofiller was 0.5 wt.% at room temperature, with the nanocomposite also showing a 33% increase in the DC breakdown strength compared to pure XLPE, similar to the results reported by Zhou et al. [64]. Based on the obtained results, Li et al. showed that a small amount of functionalized BNNS doping could significantly improve the DC breakdown strength of pure XLPE (see Figure 4a) with the introduction of deep traps in the matrix, which would easily capture the injected charges near the electrodes forming the local electric field E*_sc_* and weakening the external electric field E*_app_*, suppressing the further injection of interfacial charges, and the internal charge decreases correspondingly, as shown in Figure 4c,d. Figure 4b shows the change in the accumulated charge amount and DC breakdown strength with temperature. In the work [66,67], the influence of temperature on charge accumulation in pure XLPE was studied. It was demonstrated that the maximum charge accumulation in pure XLPE occurs in the temperature range of 50–60 °C.

Guochang Li et al. [67] also investigated the effect of BNNS concentration on the space charge accumulation characteristics of an XLPE/BNNS nanocomposite. The BNNSs’ surface was functionalized with the coupling agent KH560 in order to achieve good dispersion of nanofillers in the XLPE matrix. It was shown that the space charge in the nanocomposite could be effectively suppressed with a small amount of KH560-functionalized BNNSs (<0.5 wt.%) as a result of the deep traps that are formed in the composite. The trap levels formed in the composite were 0.95, 1.15, and 1.02 eV, respectively, for the concentrations 0.1, 0.5, and 1.0 wt.%. The trapped charge amount gradually increased with the increasing concentration of nanofiller, reaching maximum values of about 4.25 × 10^20^ m^−3^ when the nanofiller concentration was 1 wt.%. The authors argued that in small concentrations the BNNSs acted as a nucleating agent which increased crystallinity and reduced the size of crystallization [68], thus increasing the grain interface and leading to an increase in the trap number. At high concentrations (3.0 and 5.0 wt.%), the trap level in the composite was reduced, which could be due to the agglomeration of BNNSs, leading to physical shallow defects.

### 3.3. Silica-Based XLPE Insulation Materials

Silica (SiO_2_) is by far the most commonly used nanofiller in the cable insulation industry given its excellent dielectric properties, temperature stability, and mechanical strength. Chunyang Li et al. [69] investigated the influence of nano-SiO_2_ on the AC and DC electrical treeing resistance properties of pure XLPE. Based on the experiments conducted, it was demonstrated that the SiO_2_/XLPE nanocomposite with a 1.0 wt.% nanofiller contained a large number of deep traps, which inhibited the initiation and growth of the DC grounded electrical tree. Under an AC power frequency, the electrical treeing occurred faster in comparison with the DC power frequency. At an early stage of tree initiation under the AC voltage, the SiO_2_/XLPE nanocomposite inhibited the initiation and growth of electrical trees until a threshold frequency after which the growth rate of electrical trees with the nanocomposite became higher than that of unmodified XLPE. Abd Rahman et al. [70] also studied the influence of SiO_2_ nanofillers on the AC breakdown strength of XLPE. However, their work did not provide any tangible results as the AC breakdown strength of the pure XLPE and SiO_2_/XLPE nanocomposite were practically identical, which could be the result of either the agglomeration of SiO_2_ nanoparticles in the sample or the use of an incorrect methodology for analyzing the sample.

Said et al. [71] reported the use of non-functionalized and chemically functionalized silicon dioxide (SiO_2_) nanoparticles to enhance the electrical properties of pure XLPE. The functionalization of SiO_2_ nanoparticles was carried out using amino silane. The nanocomposites obtained were further subjected to relative permittivity (ε_r_) and dielectric loss (tan δ) studies. The ε_r_ values of the nanocomposites were significantly influenced by the concentration of SiO_2_ in the XLPE/SiO_2_ sample. In the case of XLPE/SiO_2_ containing a 2.0 wt.% nanofiller, the lowest ε_r_ values, especially with the SiO_2_-functionalized sample were recorded, which were about 4.1% less than that for neat XLPE at a power frequency of 50 Hz. At the same time, both the non-functionalized XLPE/SiO_2_ and amino-functionalized nanocomposites with a 5.0 wt.% nanofiller had higher ε_r_ values than neat XLPE, which was associated with the reduction in the interface interaction area between the polymer matrix and nanoparticles as a result of nanoparticle agglomeration. At the same time, the tan δ value for all the XLPE/SiO_2_ showed lower values than that of the neat XLPE specimen, while the amino-functionalized SiO_2_/XLPE samples have lower values of tan δ than non-functionalized equivalents at frequencies up to 1000 Hz, with amino-functionalized SiO_2_/XLPE containing a 5.0 wt.% nanofiller having its tan δ reduced by 86.76% in comparison to neat XLPE.

**Figure 5:**
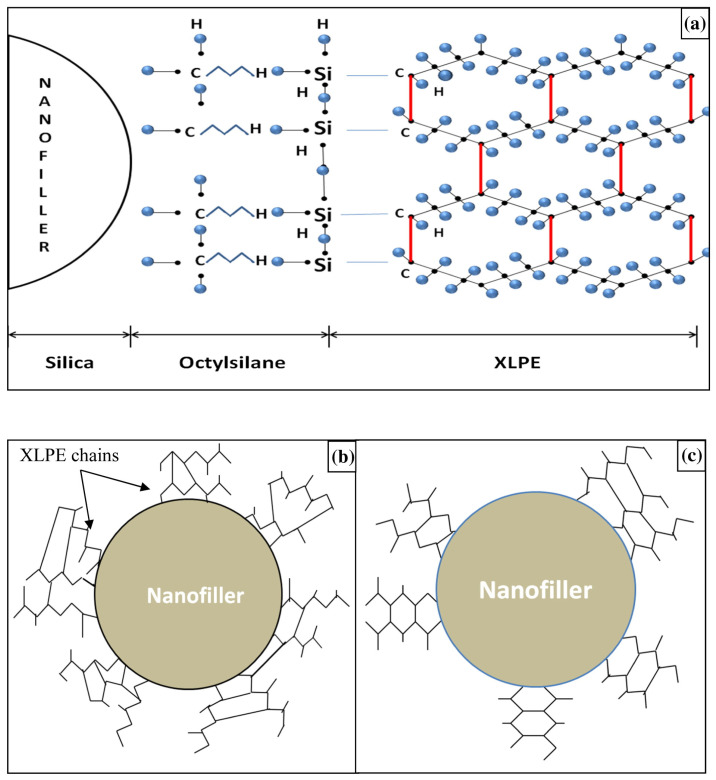
(**a**) Coupling of octylsilane-functionalized SiO_2_ into XLPE matrix; representation of polymer chain alignment with (**b**) non-functionalized and (**c**) octylsilane-functionalized SiO_2_ [72].

Sharad and Kumar [72] investigated the PD characteristics of XLPE nanocomposites with octylsilane-functionalized silica nanofillers, prepared using a twin-screw extruder and injection molding. It was reported that the octylsilane surface-modified silica/XLPE nanocomposite with a 3 wt.% nanofiller exhibited the lowest PD activity and had the highest discharge inception voltage and breakdown voltage. The improved performance of octylsilane-functionalized nanocomposites was explained with a modified polymer chain alignment model (Figure 5) suggesting that functionalized nanofillers could form a better alignment with XLPE polymer chains which enhanced the performance of XLPE nanocomposites.

In another study by Kalaivanan and Chandrasekar [73], the inception time, breakdown time, and propagation speed of electrical trees in XLPE modified with a SiO_2_ nanofiller was reported. The electrical treeing experiment data showed that the XLPE/SiO_2_ nanocomposites had a significantly higher breakdown time compared to that of pure XLPE. The improvement in the breakdown time for the nanocomposites containing 1.0 and 3.0 wt.% SiO_2_ at a 12 kV AC voltage was 12.5 and 31.25%, respectively. Meanwhile, the tree initiation time for nano-SiO_2_-filled XLPE specimens was greater than that of the pure XLPE specimen. These observations substantiated that the nano SiO_2_ particles enhanced the lifetime of the pure XLPE material.

In the work [74], the addition of organo-modified layered silicate (nanoclay) to pure XLPE was reported. The studies on the dielectric properties of the nanocomposite showed that the effective permittivity of nanocomposites increased with the increase in the concentration of nanoclay at all frequency ranges, along with the dielectric strength which also increased with the addition of the organo-modified layered silicate nanofiller, and the nanocomposite with a 5.0 wt.% concentration of nanoclay fillers resulted in a ~30% increment in dielectric strength compared to pure XLPE. Kaihao et al. [75] compared the space charge characteristics of XLPE modified with SiO_2_, functionalized with various surface modifiers: KH550, KH560, and A-151. It was shown that the nanocomposite based on SiO_2_ functionalized with KH560 (XLPE/KH560-SiO_2_) had the lowest electric field distortion rate at around 19.02% along with the uniform distribution of electric field intensity in the thick section. Di Donghe et al. [76] also studied the influence of SiO_2_ functionalization using similar surface modifiers; however, the work did not specify which SiO_2_-functionalized XLPE nanocomposite showed the best results for conductivity and DC breakdown characteristics.

Zheng et al. [77], for the first time, using density functional theory calculations, investigated the role of SiO_2_ nanofillers as a voltage stabilizer for power cable insulation. Several α-SiO_2_ nanofillers, including hydroxylated, reconstructed, doped, and oxygen vacancy top-layer defect E-SiO_2_ surface structures, were created to model the interfacial interaction of SiO_2_/XLPE nanocomposites. Based on the calculations it was shown that the SiO_2_ with incomplete hydroxy groups, oxygen vacancy defect on the top layer, and boron-doping were not suitable as potential additives due its facilitation of a H migration reaction, consequently leading to electrical tree growth in the XLPE matrix. The N-doped SiO_2_ with a completely hydroxylated surface was predicted as the most suitable additive due to its good compatibility with XLPE alongside a relatively inert nature.

Zhang et al. [78] reported an improved water tree resistance of pure XLPE upon the addition of trimethylolpropane triacrylate (TMPTA)-functionalized SiO_2_ as a nanofiller. The water tree in the case of XLPE grew emanatively around the cut defect tip, while the TMPTA-functionalized SiO_2_/XLPE nanocomposites showed a uniform water tree formation accompanied by a significantly lower growth speed, i.e., smaller size, tending toward the ground electrode. Meanwhile, the resistance to water tree growth of the non-functionalized SiO_2_/XLPE nanocomposite was not so obvious as in case of the TMPTA-functionalized SiO_2_/XLPE nanocomposites.

Said et al. [79] studied the influence of the addition of amino silane-functionalized-SiO_2_, TiO_2,_ and ZnO nanoparticles on the performance and characteristics of commercial XLPE. It was reported that the nanocomposites based on the TiO_2_/XLPE samples had their melting temperature increased by 6.85 °C in comparison with pure XLPE, but had the smallest tensile strength and elongation values. On the other hand, amino silane-functionalized SiO_2_ nanofiller-based XLPE composites showed the maximum enhancement in dielectric properties. At the same time, the presence of moisture negatively affects the electrical as well as the mechanical properties of the material. In their works Hui et al. [80,81] studied in detail the dielectric behavior of SiO_2_-modified XLPE in humid conditions. Their investigations revealed that, in comparison to virgin XLPE, the SiO_2_-modified XLPE had an increased moisture uptake. The authors postulated this to be the primary reason for the poor dielectric behavior of SiO_2_-modified XLPE nanocomposites, resulting in the formation of water shells around the nanoparticles and thereby changing the inter-particle spacing. The SiO_2_ nanoparticles contain –OH groups on their surfaces, which are hydrophilic in nature, while the XLPE, on the other hand, is a non-polar material, and barely interacts with moisture.

### 3.4. Carbon-Based XLPE Insulation Materials

Graphene nanoparticles have gained in popularity in the last 10 years due to their excellent optical transparency, high strength, and potential for tunable electronic properties. One of the commonly used form of graphene, obtained by its oxidation—graphene oxide (GO)—has unique amphiphilic nature, with both hydrophilic and hydrophobic domains, allowing it to interact with various molecules and thus making it suitable for diverse applications [82,83].

Han et al. [84] investigated the charge transport characteristics and DC breakdown properties of XLPE combined with a GO nanofiller prepared using different types of surface modifiers. It was reported that the measured current of all the nanocomposites, including neat XLPE, showed a gradual decrease with the polarization time and reached a stable state at the end of the polarization. The calculated conductivities of the pure XLPE, XLPE + 0.1 wt.% polycyclic aromatic compounds, XLPE + 0.01 wt.% untreated GO quantum wells, XLPE + 0.01 wt.% functionalized graphene oxide quantum wells, and XLPE + 0.01 wt.% functionalized graphene oxide quantum wells + 0.1 wt.% polycyclic aromatic compounds were 1.67 × 10^−16^, 6.43 × 10^−17^, 4.08 × 10^−17^, 1.44 × 10^−17^, and 2.13 × 10^−17^ S/m, respectively, indicating that the conductivity of XLPEs can be reduced by doping with polycyclic aromatic compounds and graphene oxide quantum wells. At the same time, for each of the studied samples, an exponential drop in the conductivity was observed in the first 200 s, after which the conductivity stabilizes to the above-mentioned values.

The role of the GO QWs in the XLPE matrix was studied in detail using the quantum chemistry calculation method. It was shown that the Fermi level of the XLPE matrix and GO QWs coincided with each other after contact, while considering the large specific surface area of GO QWs, a number of interfacial regions were formed in XLPE, resulting in an obvious increase in traps for both electrons and holes. At the same time, the charge transport in the interfacial region was affected by the quantum confinement effect of GO QWs. Due to the big difference in the highest occupied molecular orbital and lowest unoccupied molecular orbital gap between GO and XLPE, there is a large potential barrier for charge carrier transport for electrons and holes, explaining the mechanism by which GO QWs improve the electrical properties of XLPE [85].

In another work by the same authors [86], the effect of polarity reversal on the space charge behaviors and DC breakdown properties of on the previously prepared XLPE/GO nanocomposites was studied. It was reported that both the GO QWs and PAC inhibited the space charge accumulation in XLPE under polarity reversal. The doping of 0.01 wt.% fGO QWs into XLPEs remarkably improved the breakdown strength of the nanocomposites.

Li et al. [87] in their work studied the thermal, mechanical, and electrical properties of neat XLPE and graphene-based XLPE. It was shown that doping the XLPE matrix with small amounts (0.007–0.008 wt.%) of graphene improved its mechanical and electrical insulation properties. However, the thermal performance of XLPE saw a limited improvement upon graphene doping. Negri et al. [88] investigated the effect of GO-containing nanostructured hybrid coatings on the PD resistance capability of XLPE films and ad hoc specimens containing cavities with a controlled size and geometry. It was reported that the GO-containing nanostructured hybrid coatings not only delayed the effects of thermo-oxidation but also had a lower partial discharge rate during the second aging period, and thus indicating a better resistance towards their induced aging effect.

In another study carried out by the Han’s research group [89], the temperature dependence of the DC conductivity and space charge properties of the XLPE/GO nanocomposites was investigated. GO nanoparticles with mass fractions of 0.001, 0.01, and 0.1 wt.% were mechanically blended with the XLPE matrix. It was reported that the conductivity of the XLPE/GO nanocomposites, as well as pure XLPE, increased exponentially with temperature, while the conductivity of the nanocomposite containing 0.01 wt.% of GO was significantly lower than that of the neat XLPE.

At the same time, the DC breakdown strength of the XLPE was increased upon the addition of GO nanoparticles and, as the test temperature was increased, the breakdown strength of the samples decreased, especially for the 0.1 wt.% XLPE/GO nanocomposites. The breakdown strength of the 0.1 wt.% XLPE/GO nanocomposites was higher than that of the neat XLPE at 30 °C, while it gradually decreased when the temperature was increased to 90 °C. Du et al. [90] also carried out similar studies on the XLPE/GO nanocomposites. Their results showed that the XLPE containing a 0.01 wt% nanofiller had a lower conductivity, lower space charge accumulation, and higher DC breakdown strength than those of the neat XLPE, further confirming the results reported by Han’s group [89].

In order to understand the influence of each type of nanofiller on the dielectric breakdown strength of the virgin XLPE, a comparison is presented in Table 2 below.

The addition of metal oxides and SiO_2_ to the XLPE matrix greatly improves the dielectric breakdown strength of the nanocomposite. However, it is worth mentioning that, in comparison with graphene oxide, which requires very small loading (only 0.01 wt.%), the metal oxides as well as SiO_2_ require a relatively higher loading (around 3.5 wt.%), which could lead to the issue of nanoparticle agglomeration. At the same time, the functionalization of nanofillers with surface modifiers such as KH550, KH560, and A-151 significantly helps to address the issue of compatibility by improving the bond between the inorganic substrates and the XLPE.

## 4. Prospects in Research and Development of Next-Generation Polymer Nanocomposites for High-Voltage Cable Insulation

**Figure 6:**
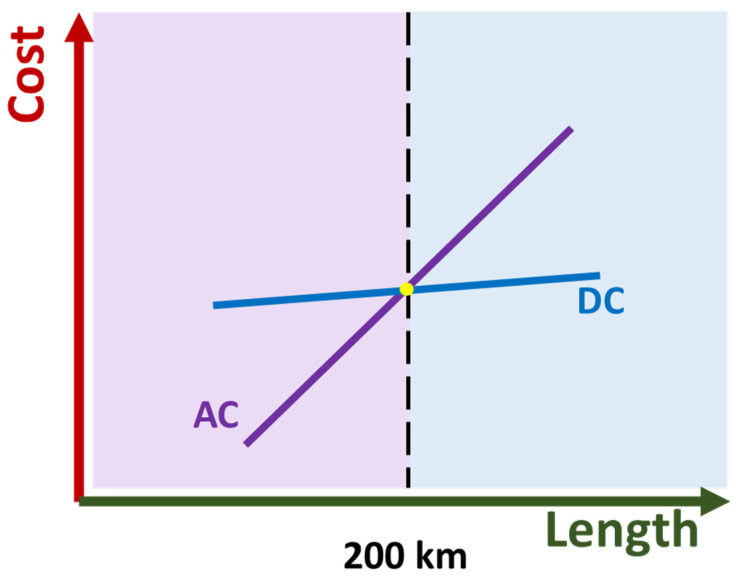
Overall cost of high-voltage AC and DC power transmission systems as a function of transmission distance.

While the XLPE remains the most popular choice for HV AC and DC cable insulation, the rise of renewable energy, especially offshore wind power generation, has created a demand for cross-sea large-capacity electric power transmission. However, HVAC cable power transmission is not suitable due the large capacitive current, which severely reduces power transmission efficiency [91]. At same time, the construction cost of HVDC transmission is lower for long distances (Figure 6) [5]. As a result, HVDC cable power transmission is considered the best choice for long-distance offshore wind power.

The process of manufacturing XLPE cables is very complicated and also results in a longer production time and higher energy consumption. In addition, the crosslinking process transforms thermoplastic polyethylene into thermoset XLPE, which is very difficult to recycle, and the crosslinking by-products of XLPE could introduce microdefects and charge accumulation under a DC electric field, causing the insulation to fail [92,93].

Polypropylene (PP) has emerged as a suitable candidate for addressing the shortcomings faced by XLPE-based HVDC cable insulation due to its superior thermal, electrical, and mechanical properties, alongside with its intrinsic high melting temperature, enabling it to carry high voltages and withstand higher working temperature, thus avoiding the use of crosslinking agents, which eliminates the degassing treatment, thereby making it a recyclable alternative to XLPE. Yoshino et al. [94] carried out a comparative study on the breakdown strength of syndiotactic PP, isotactic PP, and XLPE. The results indicate that the syndiotactic PP had a much higher breakdown strength compared to isotactic PP and XLPE. However, the high manufacturing cost of syndiotactic PP limits its range applications in cable insulation. Another commonly used approach to altering the properties of virgin PP is blending it with other thermoplastic polymers such as ethylene propylene rubber, ethylene propylene diene monomer, and polyolefin elastomers [95]. At the same time, there are several works that have reported on the incorporation of nanofillers, such as Al_2_O_3_, TiO_2_, ZnO, etc., into PP matrixes to further enhance the mechanical and dielectric properties of polymer nanocomposites [96,97,98,99,100,101]. Recently, Lee et al. [102] reported the addition of a small amount of polyvinylidene fluoride into a PP matrix as a thermoplastic voltage stabilizer. Their investigations showed an increase in DC breakdown strength by 110% and 149% at 25 and at 110 ◦C, respectively, for the PP modified with polyvinylidene fluoride compared to virgin PP. In the work [103], the authors studied the structure–performance relationship of PP modified with an elastomer and carbon black for application as cable shielding layer. While in another study [104] the potential applications of PP modified with an elastomer for cable insulation in nuclear power plants was reported. The results indicated an improvement in the AC breakdown strength by 21% for PP modified with an elastomer under the action of gamma-ray irradiation. In an interesting study carried out by Dong et al. [105], PP was grafted with styrene in order to improve its high-temperature electrical properties. Styrene-grafted PP samples showed suppressed space charge alongside with improved volume resistivity and breakdown strength at room temperature and at high temperatures.

However, research on PP-based HVDC cable insulation materials is at its initial stages and there are still key issues with PP-based materials, such as low thermal conductivity, space charge accumulation, and aging under high temperatures and DC stresses.

## 5. Conclusions

XLPE-based polymer nanocomposites have been the cornerstone of the development of modern dielectric insulation materials for high-voltage cable insulation. The present review discusses the influence of the addition of various types of nanofillers on the mechanical and electrical properties of XLPE. At the same time, the surface modification of nanofillers and preparation methods of XLPE-based nanocomposites are reviewed in detail. Various nanodielectric models explaining the structure and properties of the interface between the polymer matrix and nanofiller have been discussed. It has been shown that various parameters, including nanofiller loading, type, surface chemistry, morphology, and temperature, have substantial influence on the dielectric properties of XLPE nanocomposites. While overcoming certain critical problems related to the compatibility between the polymer matrix and the nanoparticle, morphological changes, aggregation and agglomeration, and erosion still remain challenges and addressing them is crucial in terms of enhancing the efficiency of XLPE-based polymer nanocomposites.

What does the future hold?

While XLPE is projected to be the primary choice of insulation for high-voltage cables for the foreseeable future, as the global economy transitions to renewable energy sources, environmental concerns are becoming strong components to be taken into account when selecting insulation materials and, given the fact that XLPE is not easily recycled and has a complex manufacturing process, traditional thermoplastics such as polypropylene and nanocomposites based on it are likely to dominate future research. Such ambitious tasks can easily be considered the ‘holy grail’ and, if the XLPE wants to maintain its edge over other polymers as the primary material for high-voltage cable insulation, a major breakthrough in the development of XLPE-based nanocomposites is required.

## Figures and Tables

**Table 1:** Dielectric properties of modified and pure XLPE nanocomposites [54].

	Modifier Loading (wt.%)	Breakdown Strength (kV/mm)	Change in Breakdown Strength (%)	Relative Permittivity (ε_r_)	Loss Factor, (tanδ)
Pure XLPE	0	31.23	-	2.91	0.0113
XLPE/non-functionalized ZnO	0.5	25.00	−19.95	3.32	0.0617
XLPE/functionalized ZnO	0.5	32.96	+5.54	3.17	0.0403
	2.0	34.01	+8.19	2.79	0.0339
	3.5	34.16	+9.38	2.84	0.0285
	5.0	31.62	+1.24	2.93	0.0226

**Table 2:** Influence of different types of nanofillers on the in dielectric breakdown strength of virgin XLPE.

No	Nanofiller	Loading (% wt.)	Change in Dielectric Breakdown Strength with Respect to Virgin XLPE (%)	Reference
1	Al_2_O_3_	1.0	–	[49]
2	Al_2_O_3_ functionalized with hybridized layered double hydroxide	0.8	+15.6 AC	[50]
3	ZnO	0.5	−19.9 AC	[54]
4	ZnO functionalized with γ-Aminopropyltriethoxy silane	3.5	+9.4 AC	[54]
5	TiO_2_ functionalized with KH560	0.5	+13.5 DC	[60]
6	*h*-BN	0.1	+39.0 DC	[63]
7	BN nanosheets functionalized with KH550	0.5	+33.0 DC	[65]
8	SiO_2_	2.0	+2.5 AC	[71]
9	SiO_2_ functionalized with γ-Aminopropyltriethoxy silane	2.0	+29.6 AC	[71]
10	Functionalized graphene oxide quantum wells	0.01	+33.3 DC	[84]
11	Graphene oxide	0.01	+17.1 DC	[89]

## Data Availability

No new data were created or analyzed in this study. Data sharing is not applicable to this article.
